# Reproductive development in *Trithuria submersa* (Hydatellaceae: Nymphaeales): the involvement of *AGAMOUS*-like genes

**DOI:** 10.1007/s00425-024-04537-5

**Published:** 2024-09-26

**Authors:** Silvia Moschin, Sebastiano Nigris, Elisabetta Offer, Nicola Babolin, Adriana Chiappetta, Leonardo Bruno, Barbara Baldan

**Affiliations:** 1 Department of Biology, University of Padova, Padua, Italy; 2 Botanical Garden of Padova, University of Padova, Padua, Italy; 3 Department of Biology, Ecology, and Hearth Sciences (DiBEST), University of Calabria, Arcavacata Di Rende, CS Italy; 4 National Biodiversity Future Center, Palermo, Italy; 5 Department of Biosciences, University of Milano, Milan, Italy

**Keywords:** *Trithuria submersa*, Hydatellaceae, MADS-box genes, Early-diverging angiosperms, Flower development

## Abstract

**Main conclusion:**

In the early diverging angiosperm* Trithuria submersa* *TsAG1* and *TsAG2* are expressed in different flower organs, including bracts, while* TsAG3* is more ovule-specific, probably functioning as a D-type gene.

**Abstract:**

Species of *Trithuria*, the only genus of the family Hydatellaceae, represent ideal candidates to explore the biology and flower evolution of early diverging angiosperms. The life cycle of *T. submersa* is generally known, and the “reproductive units” are morphologically well described, but the availability of genetic and developmental data of *T. submersa* is still scarce. To fill this gap, a transcriptome analysis of the reproductive structures was performed and presented in this work. This analysis provided sequences of MADS-box transcription factors, a gene family known to be involved in flower and fruit development. In situ hybridization experiments on floral buds were performed to describe the spatiotemporal expression patterns of the *AGAMOUS* genes, revealing the existence of three *AG* genes with different expression domains in flower organs and in developing ovules. *Trithuria* may offer important clues to the evolution of reproductive function among early angiosperms and Nymphaeales in particular, and this study aims to broaden relevant knowledge regarding key genes of reproductive development in non-model angiosperms, shaping first flower appearance and evolution.

**Supplementary Information:**

The online version contains supplementary material available at 10.1007/s00425-024-04537-5.

## Introduction

Current botanical research shows growing interest in extant plant species that trace back their ancestry to the early angiosperms. Among the early-diverging extant angiosperm lineages, *Trithuria*, the only genus of the Hydatellaceae family (Sokoloff et al. 2008), is particularly interesting since its phylogenetic position has been quite recently reconsidered. Indeed, this family was transferred from their former placement in the monocots, close to the grasses, to the water-lily order Nymphaeales (Saarela et al. 2007). The genus *Trithuria* currently comprises less than 20 species (Iles et al. 2012, 2014; Sokoloff et al. 2019). They are small and inconspicuous plants that received little attention from botanists prior to their taxonomic reassignment.

In contrast with almost all other early-diverging angiosperms, species of the genus *Trithuria*, due the small habit, annual life cycle, and the capacity for self-fertilization in most species (Taylor et al. 2010; Sokoloff et al. 2011) represent ideal candidates as a model system for studying the biology and evolution of the first branches of angiosperm phylogeny (Vialette-Guiraud et al. 2011; Povilus et al. 2015).

*Trithuria submersa* is a species found in seasonal, rain-fed wetlands of southwestern Western Australia and parts of southern New South Wales, South Australia, Victoria, and Tasmania (Sokoloff et al. 2008). Plants germinate early in the winter as the dried-out habitats become sufficiently wet, grow vegetatively during winter and initiate inflorescences in spring, which may proceed to a full development prior to the plants become exposed as the water recedes through evaporation. *T. submersa* plants tend to grow in shallow water that submerges them for a relatively short period during winter, or they may even be exposed and grow only in saturated mud (Sokoloff et al. 2011).

In vitro cultivation of *T. submersa* has been recently developed (Kynast et al. 2014), thus allowing setting up a deeper investigation of its biology. To date, studies of *T. submersa* have mostly concentrated on characterizing vegetative and reproductive morphologies, but also some ecological and genomic aspects (Rudall et al. 2008, 2009a, 2009b; Taylor et al. 2010; Prychid et al. 2011; Sokoloff et al. 2013).

Rudall and colleagues in 2007 described well the structure of the “reproductive unit”. They are bisexual, presenting stamens in a central position, surrounded by many carpels that comprise most of the structure. Each carpel exposes several unusual uniseriate stigmatic hairs and bears a single apical ovule. Each reproductive unit is protected by some involucral bracts. The fruit is markedly triquetrous, with three prominent ribs, and contains one single ovoid seed.

Molecular data of *Trithuria submersa* are still scarce, as they mostly regard sequences of few molecular markers for taxonomy. Marques et al. (2016) studied the genetic variation in populations of *Trithuria submersa* across Australia by performing a transcriptome-derived microsatellite analysis. Their study highlights the presence of two deep lineages of *T. submersa* that are geographically separated by the Nullarbor Plain (AUS), and that might have experienced different polyploidization events. Besides this work and a chromosome study performed by Kynast et al. (2014), genetic information such as gene and protein sequences are very limited. Among important genes for plant reproductive development, MADS-box genes are well known and have been studied in many seed plants. There are more than a dozen clades of MADS-box genes in angiosperms, of which those with functions in the specification of floral organ identity have been especially characterized (Gramzow et al. 2023). Several clades of MADS-box genes had been established before the diversification of flowering plants (Münster et al. 1997), for example the *AGAMOUS* gene family, which has been studied in many gymnosperms (Jager et al. 2003; Zhang et al. 2004; Lovisetto et al. 2012; D’Apice et al. 2022), characterizing *AGAMOUS* as one of the essential genes for the development of the reproductive structures of seed plants. In angiosperms, *AGAMOUS* subfamily members typically promote stamen and carpel identity and floral meristem determinacy (Kramer et al. 2004). *AGAMOUS* expression in core eudicots is generally regulated to be confined to stamen and carpel whorls, according to the ABC flower development model, while outside eudicots the situation can vary, and has been correlated with different flower morphologies and architectures (Theissen and Melzer 2007). In some early-diverging angiosperms, *AGAMOUS* genes have been found expressed also in perianth elements, such, for example in *Nymphaea odorata* (Yoo et al. 2010) and in *Nymphaea caerulea* (Moschin et al. 2021), where two *AG*-like genes showed a broad expression profile in flower organs. This broader pattern of expression has been also found for other MADS-box genes in “basal” angiosperms, for example in *Amborella*, *Illicium* and *Nuphar* (Buzgo et al. 2004; Kim et al. 2005), supporting the “fading borders model” of ABC flower organ identity genes for “basal” angiosperms (Buzgo et al. 2004; Soltis et al. 2007, 2009; Theissen and Melzer 2007). A deep investigation of the MADS-box gene expression in other Nymphaeales species can add novel data and contribute to this discussion. The flower units of *Trithuria submersa* are characterized by numerous carpels, which comprise most of the structure. Given this morphological feature, the remarkable phylogenetic position among early-diverging angiosperms, and the paucity of deposited molecular data, we focused this study on MADS-box genes expressed during *Trithuria submersa* flower development, paying particular attention to the *AGAMOUS* gene subfamily. We cultivated *T. submersa* in vitro and set up an RNA-sequencing of its floral buds, with the primary purpose of identifying MADS-box players involved in the formation of its reproductive units. Morphological observation of its tiny reproductive structures accompanied the molecular study. We then performed in situ hybridization experiments on floral buds, with the purpose of describing carefully the spatiotemporal expression patterns of the *AGAMOUS* genes identified.

*Trithuria* may offer important clues to the evolution of reproductive function among early angiosperms and Nymphaeales sensu lato in particular, and this study aims to contribute to extending relevant knowledge regarding key genes of reproductive development in non-model angiosperms.

## Materials and methods

### In vitro cultivation of* Trithuria submersa*

The seeds of *Trithuria submersa* Hook.f. used for this work are from the National Herbarium of Victoria (Melbourne, Australia) and were collected by Prof. Neville Walsh (voucher number MEL 2388684). Location of the collection is 37° 03′ 33″ S 142° 04′ 07″ E. Alt.:205 m.

*Trithuria submersa* seeds were sterilized by ethanol (1 min in 70% EtOH 0,05% Triton X-100, 1 min in 100% EtOH). After the treatment, seeds were allowed to air dry on sterile paper. Once dry, seeds were soaked in deionized water added with 5 ppm GA3 (120 rpm of shaking, overnight at room temperature, Rudall et al. 2009b), then sowed in ½ MS medium with 2% sucrose, solidified with 0.7% w/v agar–agar. Plates have been incubated in the dark at 13 °C until seed germination. Seeds took three/four weeks to germinate. Seedlings were then transferred in Magenta boxes (200 mL) with ½ MS medium solidified with 0.7% w/v agar–agar and grown at 23 °C with a photoperiod of 16 h light/8 h dark (Kynast et al. 2014). *Trithuria submersa* develops its reproductive structures (tiny floral buds enclosed by a few bracts) after about three months of in vitro cultivation. The reproductive structures were sampled before (closed buds) and after anthesis (open buds).

### RNA extraction, purification, and quantification

Flower buds before anthesis were collected from ten different plants, immediately frozen in liquid nitrogen, and stored at −80 °C until procession for RNA extraction. Total RNA was extracted using the protocol described by Lovisetto et al., (2012), quantified using a NanoPhotometer® (IMPLEN), and treated with DNase I (NEB—New England Biolabs®) to remove genomic DNA. DNase I has been removed through the RNA Clean & Concentration™-5 kit (Zymo Research). RNA has been quantified again by Qubit 4 Fluorometer (Thermo Fisher Scientific) and then prepared for RNA-sequencing, according to the instruction of the sequencing company (Novogene, HK).

### RNA-sequencing of young flower buds

The RNA-sequencing was performed by Novogene (Hong Kong). Illumina sequencing generated 150 bp long paired-ended reads. Raw reads were quality filtered and then de novo assembled using the Trinity software (Grabherr et al. 2011) to obtain the transcriptome dataset (SRR29709627). FASTA sequences of transcripts were also analysed by the online tool TRAPID (http://bioinformatics.psb.ugent.be/trapid_02/) to better identify MADS-box genes of MIKC^C^-type. The transcripts of interest were isolated, and their annotation was confirmed by BLASTn alignments. MADS-box gene sequences identified in this work have been deposited in the NCBI GenBank database, and Accession Numbers are provided in Table 1.

**Table 1:** MADS-box transcripts isolated in *Trithuria submersa.* The NCBI accession number is provided for each transcript

Gene subfamily	Identified gene in *Trithuria submersa*	NCBI accession number
*AGAMOUS*	*TsAG1*	PP885410
	*TsAG2*	PP885411
	*TsAG3*	PP885412
*PISTILLATA*	*TsPI-1*	PP885413
	*TsPI-2*	PP885414
*APETALA3*	*TsAP3*	PP885415
*B*_*sister*_	*TsB*_*sister*_	PP885416
*AGL6*	*TsAGL6*	PP885417
*SEPALLATA*	*TsSEP*	PP885418
*AGL15*	*TsAGL15*	PP885419
*JOINTLESS*	*TsJNT*	PP885420
*SOC*	*TsSOC*	PP885421
*AGL12*	*TsAGL12*	PP885422

### Phylogenetic analysis of *Trithuria* AGAMOUS sequences

A dataset of AGAMOUS-, SHATTERPROOF/PLENA- and SEEDSTICK-*like* protein sequences has been constructed from the NCBI database. The alignment has been done by MAFFT (https://mafft.cbrc.jp/alignment/server/index.html) and then uploaded to raxmlGUI 2.0 (Edler et al. 2021) to perform a Maximum Likelihood phylogenetic analysis. The program integrates RAxML-NG and ModelTest-NG for phylogenetic analysis. After finding the best substitution model, the tree has been run with 100 bootstrap replicates.

### Sample fixation, embedding, and sectioning

Fresh samples (both closed and open floral buds) were fixed in a 4% paraformaldehyde solution in 1X Phosphate Buffered Saline (PBS) (10X PBS = 1.3 M NaCl, 70 mM Na_2_HPO_4_, 30 mM NaH_2_PO_4_; pH 7.0) with vacuum infiltration, and maintained in fixative overnight at 4 °C. Samples were then washed with 1X PBS (two times, 30 min each wash), and dehydrated using an ethanol series (30%, 50%, 70%, 85%) for one hour each step, followed by 95% ethanol overnight, and finally, two 100% ethanol stages for 30 min each. After the dehydration step, ethanol 100% was gradually replaced with xylene (1:3; 1:1; 3:1; 4:0; 4:0 xylene:ethanol, for 1 h each). Then, samples were embedded in Paraplast Plus (Sigma-Aldrich, Milan, Italy). *Trithuria* samples were maintained at 4 °C until they were processed.

### Light microscopy observation

Sections of 8–10 µm were cut with the microtome Leica RM 2125 RT. Slides were deparaffinised, rehydrated, and stained with 0.5% (w/v) toluidine blue in distilled water, and mounted with Canada balsam. Pictures of transverse sections were acquired with a Leica DM500 Microscope equipped with a Leica ICC50W digital camera (Leica Biosystems, Milan, Italy).

### In situ hybridization of *AGAMOUS* genes

RNA probes for in situ hybridization were designed on the target gene sequences (Supplementary Table 1) and in vitro synthetized from polymerase chain reaction (PCR)‐derived DNA templates as explained below. DNA templates were amplified from cDNA samples by Wonder Taq Polymerase (EuroClone, Milan, Italy), using gene-specific primers listed in Supplementary Table 1. PCR products were purified with the PureLink PCR Purification Kit (Invitrogen, Waltham, Massachusetts, USA). RNA Digoxigenin (DIG)‐labeled antisense and sense probes were synthetized from purified PCR amplicons by using T7 RNA polymerases (Roche, Rotkreuz, Switzerland) according to the manufacturer’s protocol. The reaction mix contained the DIG RNA labeling mix (Roche, Rotkreuz, Switzerland) and the RNase inhibitor RNaseOUT (Invitrogen, Waltham, Massachusetts, USA). We used the hybridization procedure described by Ambrose et al. (2000). Probe hybridization was performed at 55 °C overnight in a 50% formamide‐saturated box. The antibody Anti‐Digoxigenin‐AP Fab fragments (Roche, Rotkreuz, Switzerland) were diluted 1:700 and incubated for 90 min at room temperature, and the detection with the NBT/BCIP chromogen (Promega, Madison, Wisconsin, USA) was performed overnight. After stopping the reaction, the slides were dehydrated, dried, and permanently mounted using Entellan New (Merck, Darmstadt, Germany). Slides were observed and photographed with a Leica DM500 optical microscope (Leica Biosystems, Milan, Italy).

## Results

### Reproductive structures of *Trithuria submersa*

**Fig. 1:**
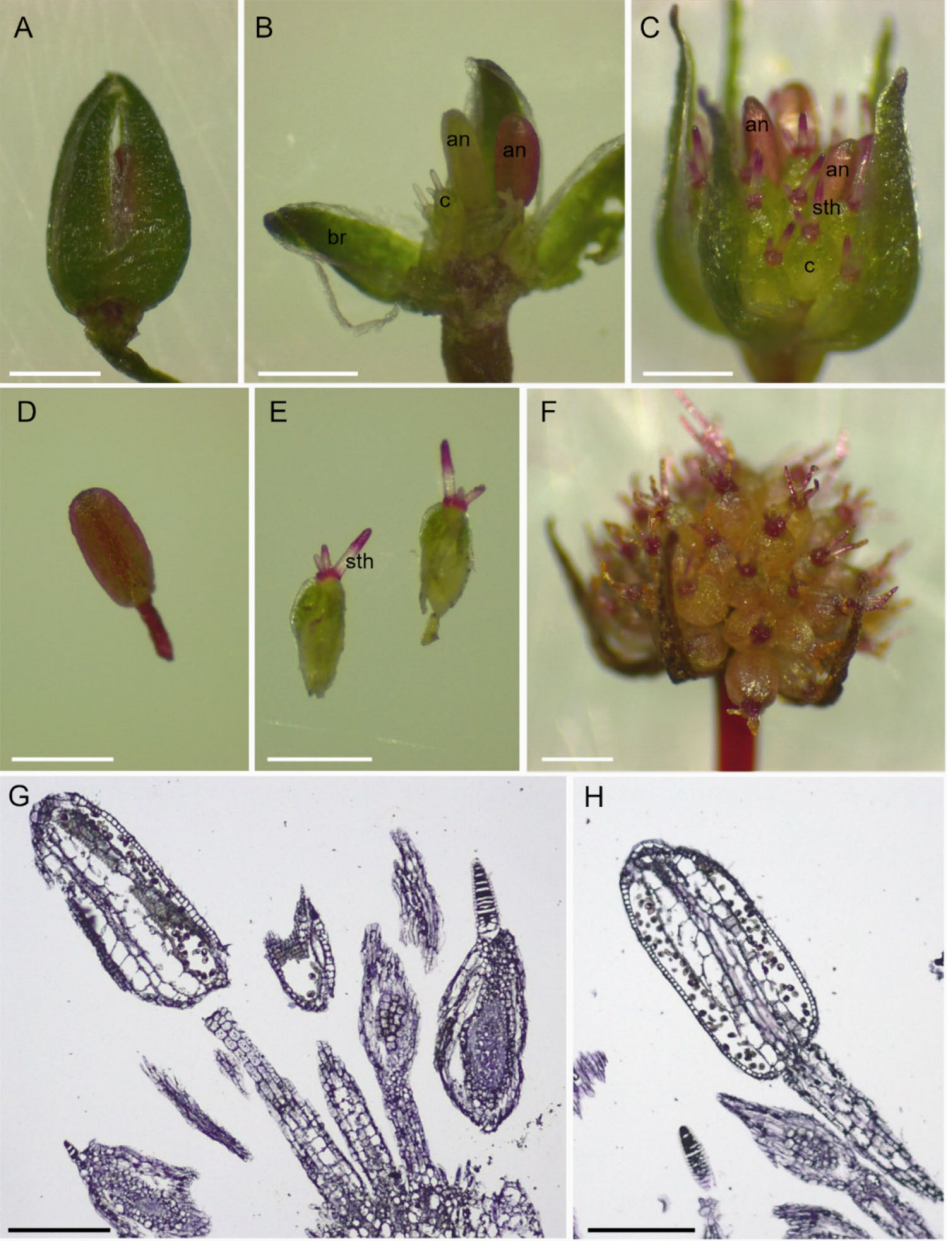
Reproductive structures of *Trithuria submersa*. **A** Pre-anthetic flower bud (stage 1). **B** Pre-anthetic flower bud (stage 1) opened under the stereomicroscope to show the reproductive organs. Anthers (an) are at two stages of development: the younger is yellow-like, while the older has developed a mature red-like coloration. **C** Flower bud at anthesis (stage 2). **D** Stamen at stage 2. **E** Carpels at stage 2. Stigmatic hairs (sth) at the apex of the carpels. **F** Flower bud in senescence with immature fruits (and seeds). **G** Paraffin-embedded section stained with toluidine blue to show the structure of stamens and carpels. **H** Paraffin-embedded section stained with toluidine blue showing mature anther structure. Scale bars in **A**–**F**: 0,5 mm; scale bars in G and H: 0,2 mm

When the plant of *Trithuria submersa* is sexually mature, it begins to produce several peduncles that bear at their apex the bisexual reproductive structures, consisting of single and small flower buds enclosed by a few bracts. Reproductive structures were primarily observed at three stages of development (Fig. 1). The first consisted of pre-anthetic flower buds (Fig. 1A, B), the second of post-anthetic buds (Fig. 1C, D, E), and the third stage corresponded to senescing buds, some of which contained developing seeds (Fig. 1F). The various stages can be recognized by the length of the peduncle: it gets longer as the plant reaches the stage of reproductive maturity. Observation of several plants allowed us to identify buds with a variable number of bracts (4 to 6), carpels (20–30), and stamens (2–3).

The structures enclosed in pre-anthetic buds (stage 1) are still immature. The bracts, well adherent to each other, show a dark green coloration, with a semi-transparent edge (Fig. 1A, B). Once the bud is opened, it is possible to observe the structure of the anthers, which clearly emerge in a central position, surrounded by numerous carpels. In agreement with Rudall et al. (2007), we observed that stamens are generally at different stages of development. In particular, one of them already has a reddish-brown anther, a colour that is maintained even post-anthesis, whereas the younger anther becomes pigmented later (Fig. 1B). At this stage of development, the stigmatic hairs in the apical position of the numerous immature carpels do not present the scarlet colour that they show at maturity (Fig. 1B).

Figure 1C shows floral buds at anthesis (stage 2). The stamens are centrally located and surrounded by several carpels. Their coloured anthers are raised above the carpels.

Phenotypic transitions characteristic of carpel-fruit and ovule-seed transformation were observed at stage 3 (Fig. 1F). The entire structure is more open, almost forming a hemisphere, and this is because the ovary has enlarged allowing the formation of seeds within the various carpels. At this stage, the bracts are withered, and the fruits show a yellowish coloration (Fig. 1F). Carpels with ovules inside are clearly visible in Fig. 1G, where a flower section is shown. In Fig. 1H a stamen section shows the structure of the anthers with pollen grains inside them.

### MADS-box genes of MIKC^C^-type identified by RNA-sequencing

The RNA-seq provided 43 transcripts that were annotated as sequences coding for MADS-box transcription factors of MIKC^C^-type. From all these sequences, 10 different types of gene families were identified (Table 1). The sequencing of different transcripts ascribed by similarity to single genes could be due to alternative splicing events. Table 1 summarizes the MIKC^C^-type MADS-box genes identified in this study and deposited in the NCBI database.

### The three *AGAMOUS* genes of *Trithuria submersa*

**Fig. 2:**
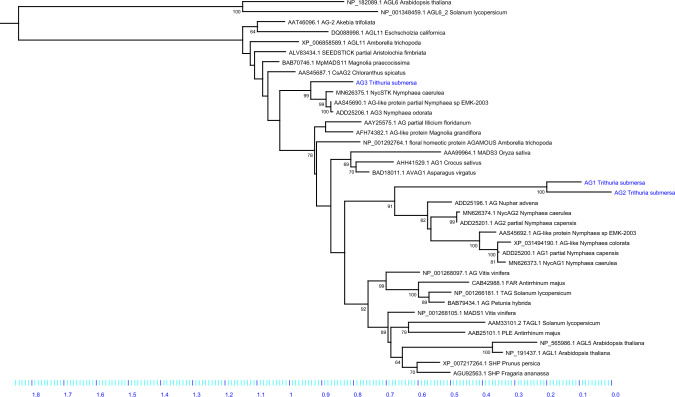
Phylogenetic analysis of the three AGAMOUS of *Trithuria submersa*. Maximum Likelihood phylogenetic analysis of *Trithuria* AGAMOUS protein sequences performed by raxmlGUI 2.0. TsAG1 and TsAG2 fell in the group of AGAMOUS-like proteins of Nymphaeales, while TsAG3 fell into the AGL11/SEEDSTICK group

In this study, we focused on the characterization of genes belonging to the *AGAMOUS* subfamily. The sequencing of mRNAs present in young flower buds of *Trithuria* allowed us to identify three different *AG-*like transcripts. A phylogenetic study of their amino acid sequences (Fig. 2) showed differences between them. TsAG1 and TsAG2 are more similar to each other and fall in the group of AGAMOUS-like proteins of Nymphaeales, while TsAG3 falls into the AGL11/SEEDSTICK group of proteins (Fig. 2).

### Expression analysis of *TrithuriaAGAMOUS* genes by in situ hybridization

To improve the identification and characterization of these three genes, we analysed their expression domains in the reproductive structures of *Trithuria* by in situ hybridization experiments. The study was performed on two stages of development: stage 1 (pre-anthetic buds) and stage 2 (post-anthetic buds) (Fig. 1).

**Fig. 3:**
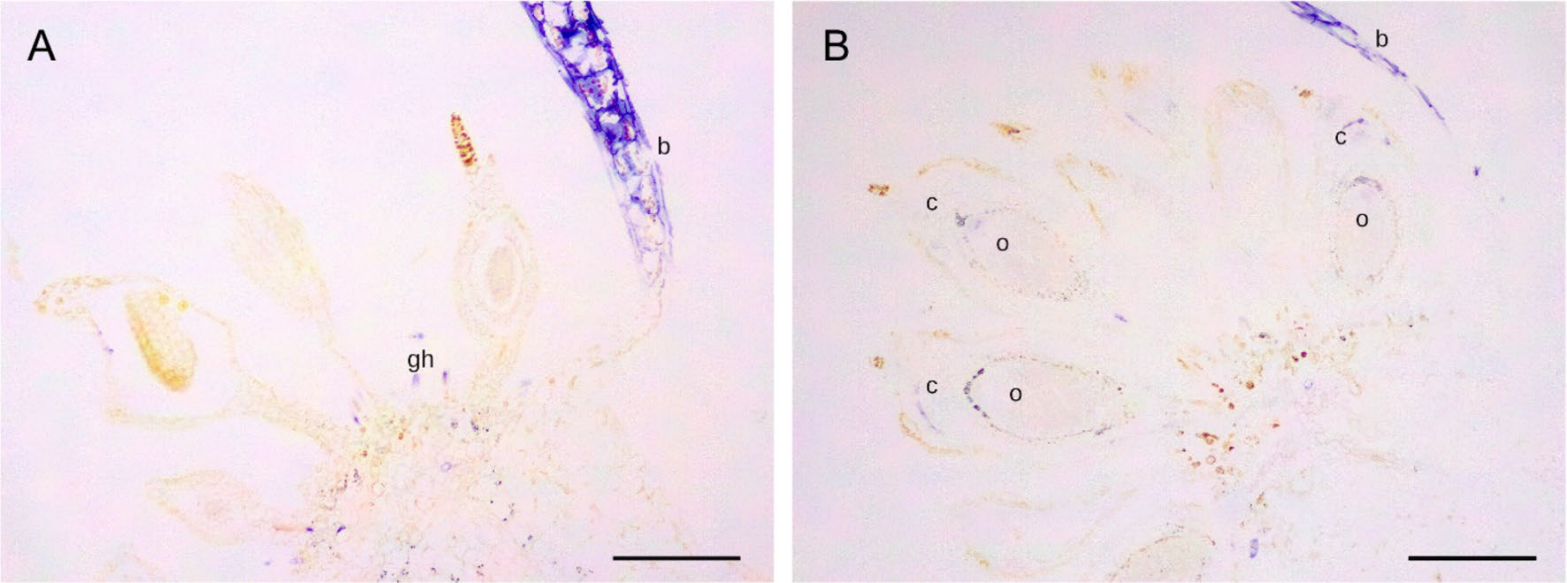
Gene expression analysis of *TsAG1* by in situ hybridization experiments. The purple-blue staining indicates the expression of the gene. **A** *TsAG1* is expressed in bracts (b). A faint expression has been detected in the tip of the glandular hairs (gh) at stage 1. **B** At stage 2 of development, *TsAG1* expression is slightly visible in the apical portion of the ovule (o) and the carpel (c). Scale bars: 0,2 mm

**Fig. 4:**
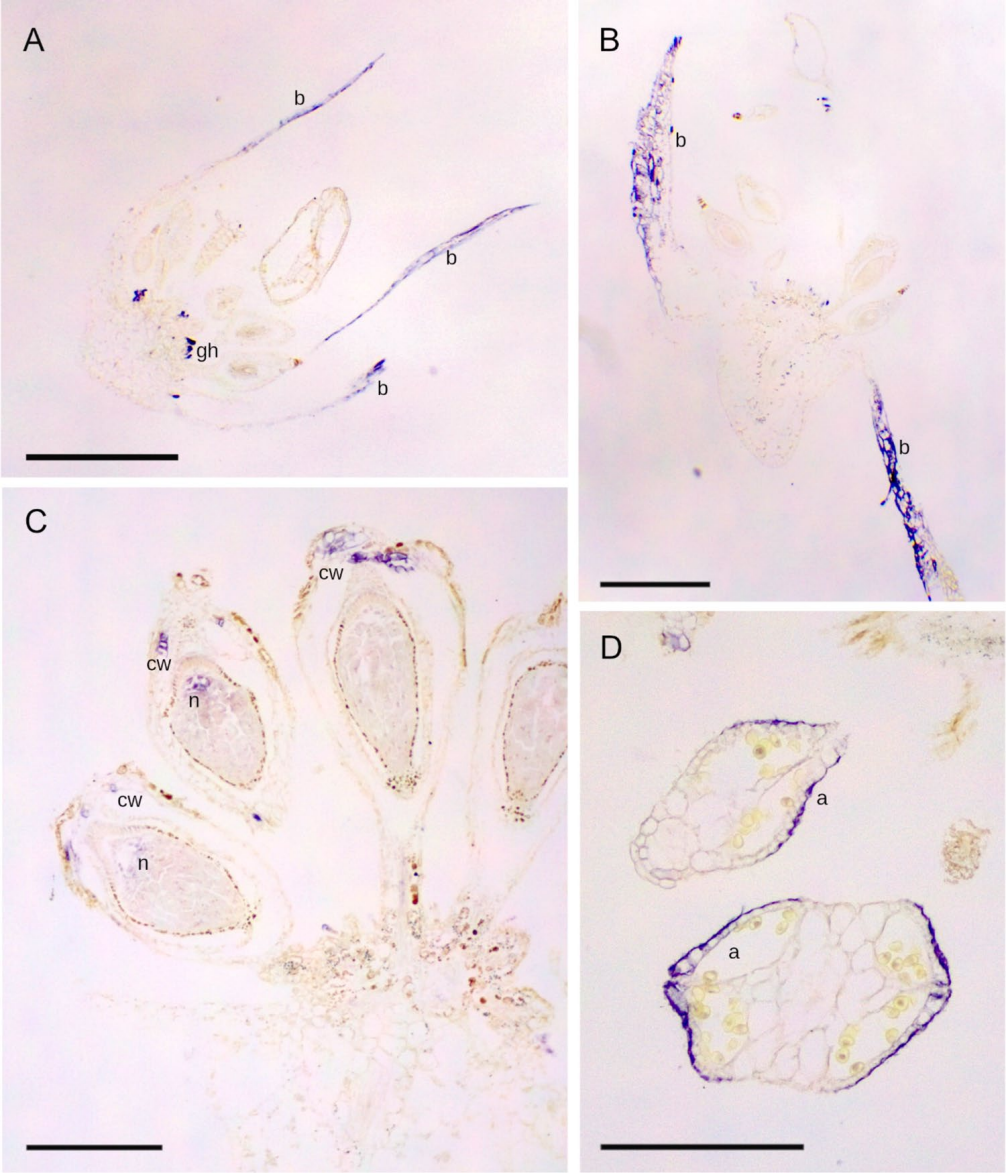
Gene expression analysis of *TsAG2* by in situ hybridization experiments. The purple-blue staining indicates the expression of the gene. **A** and **B** At stage 1, *TsAG2* is expressed in bracts (b) and in the tip of the glandular hairs (gh). **C** At stage 2 of development, *TsAG2* expression is detected in the apical portion of the carpel wall (cw) and in the ovule, in the nucellus surrounding the embryo-sac (n). **D** The expression of *TsAG2* in two cross-sections of mature anthers (a) is visible in the epidermal cells (arrowhead). Scale bar in A and B: 0,5 mm; scale bar in **C** and **D**: 0,2 mm

*TsAG1* and *TsAG2* showed a similar expression pattern, presented in Figs. 3 and 4. At the early stage of development (stage 1), their expression has been detected in the bracts (Figs. 3A, 4A and B). No signal was clearly evident at the level of stamens and pistils, nor in young ovules, but they showed expression in the tip of the glandular hairs (Figs. 3A, 4A). Later in flower development, the expression of *TsAG1* and *TsAG2* was mostly located at the apical part of the ovule, in the nucellus surrounding the embryo sac (Figs. 3B, 4C), with *TsAG2* showing a more intense signal (Fig. 4C). A clear expression pattern of *TsAG2* was well visible in mature cross-sectioned anthers, where the signal was located in their epidermal cells (Fig. 4D).

**Fig. 5:**
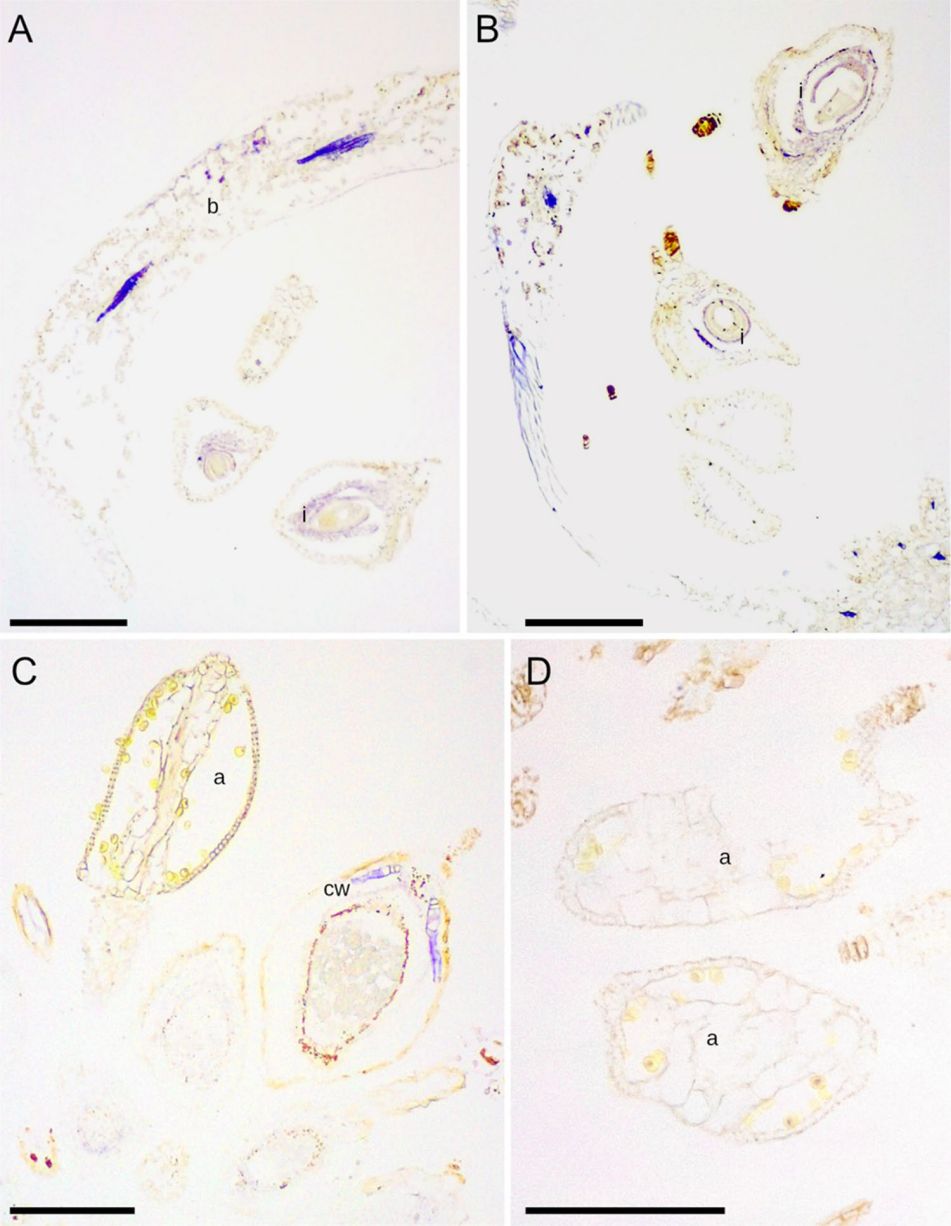
Gene expression analysis of *TsAG3* by in situ hybridization experiments. **A** and **B** At stage 1, the expression of *TsAG3* is detected in ovule integuments (i). A staining is visible also at the level of vasculatures inside bracts (b). **C** At stage 2, the expression was more evident in the ovary, restricted to the distal part of the carpel wall (cw). **D** *TsAG3* expression is not detected in *T. submersa* anthers (a). Scale bars:0,2 mm

The expression of *TsAG3* has been mainly found in female tissues (Fig. 5). More precisely, at stage 1, *TsAG3* was found in ovule integuments (Fig. 5A, B), while at stage 2, the signal was more evident in the ovary, restricted to the distal part of the carpel wall (Fig. 5C). Figures 5C and D show the absence of *TsAG3* expression in anthers.

**Fig. 6:**
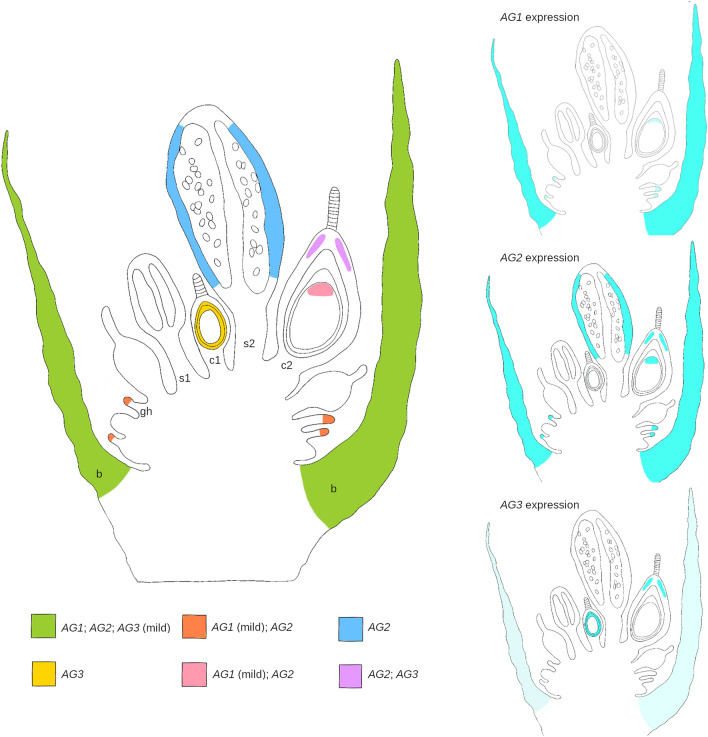
Diagram summarizing the expression domains of *TsAG1*, *TsAG2* and *TsAG3*. *TsAG1* and *TsAG2* share most of the expression pattern, with *TsAG2* more evident in the reproductive organs. The expression of *TsAG3* is quite concentrated in female structures (ovule integuments and apical portion of the carpels). b = bract; gh = glandular hair; s = stage 1 stamen; c1 = stage 1 carpel; s2 = stage 2 stamen; c2 = stage 2 carpel

Figure 6 summarizes the expression data obtained by in situ hybridization experiments, highlighting both similarities and differences between the three expression domains. Our analysis pinpointed that *TsAG1* and *TsAG2* are both expressed in bracts and share a similar expression pattern in female structures, with *TsAG2* more expressed. The latter has been also found in mature anthers. *TsAG3* has been mainly found in young ovules, resulting in agreement with what came out from the phylogenetic analysis (*TsAG3* can be an *STK*-like gene).

## Discussion

### The interpretation of *Trithuria* flower structures is still an open discussion

*Trithuria submersa* has bisexual reproductive units that terminate peduncles whose length is correlated with the age of the reproductive unit. We observed that a single unit is made up of four (sometimes five or six) involucral perianth-like bracts and several pistils surrounding two or three stamens. These observations are consistent with the description by Rudall et al. (2007). The authors also observed the initiation of the development of the reproductive organs. Bracts form two whorls that enclose carpels and stamens. Carpels are initiated centrifugally, and stamens emerge in an almost central position. At an early stage, stamens differ markedly in size and development (Rudall et al. 2007). This asynchrony of development between stamens has also been shown in this work. Stamens usually open in succession, anthers are abscised following anthesis, but the filaments remain attached to the reproductive structure.

Each pistil in Hydatellaceae is morphologically and developmentally consistent with a solitary ascidiate carpel, and each carpel bears a single apical ovule (Rudall et al. 2007). Other early-divergent clades of angiosperms also have ascidiate carpels with a single median ovules, or only a few ovules (Endress and Igersheim 2000). According to Endress and Doyle (2015), carpels with a single median pendent ovule are probably plesiomorphic in angiosperms. *Trithuria submersa* pistils lack a style and bear several apical stigmatic hairs. The sequence of carpel initiation is centrifugal, and carpel and stamen arrangement cannot be described as whorled, spiral, or even chaotic, but rather follows zigzag patterns (Rudall et al. 2007 and our observations).

The fruit is triquetrous, with three prominent ribs. At dehiscence, the ribs curve and separate from the rest of the pericarp, but they remain attached to the distal part of the fruit (Rudall et al. 2007; Sokoloff et al. 2013; our observations). Indeed, in the natural habitat, mature seeds can be mainly found partially enclosed in the fruit structure.

The morphological analysis of *Trithuria* floral structures has evidenced their peculiarities, making controversial to even define them as “flowers”. Although the bract-like phyllomes that enclose the buds show similarities with the perianth organs in flowers, Rudall et al. (2009a) stated that the reproductive units of Hydatellaceae cannot be regarded as “typical” flowers because the arrangement of the reproductive organs (pistils and stamens) is not whorled and multiple carpels develop centrifugally surrounding the stamens. The same authors also showed the limitations of an inflorescence interpretation for these structures. Indeed, if each reproductive unit could represent a highly specialized aggregation of extremely reduced unisexual flowers, it means that these structures are morphologically very different from those of other Nymphaeales (Rudall et al. 2007).

Rudall et al. (2009a) described a *Trithuria* reproductive unit as a “nonflower”: a structure that contains typical angiosperm carpels and stamens but does not allow recognition of a typical angiosperm flower. The authors stated that this term could combine cases of secondary loss of flower identity and cases of a pre-floral condition.

However, the interpretation of extant *Trithuria* floral structures remains open to discussion because of the difficulties of reconstructing their ancient aspect and the evolutionary steps made by their floral morphologies. Evolutionary-developmental genetics studies of early-diverging angiosperms may provide clues to this topic; for instance, a comparative analysis of MADS-box floral organ identity genes in *Trithuria*, *Hydatella* and other species belonging to the Nymphaeales order can be useful.

### Many different MADS-box gene subfamilies have been identified in the reproductive units of *Trithuria submersa*

MADS-box genes are well known for their involvement in nearly all aspects of plant development (Gramzow and Theißen 2010, 2013). A subset of them specifies different floral organ identities acting as homeotic selector genes, a process of key importance for plant reproductive development. Extensive genetic research conducted with eudicot model systems has provided a comprehensive understanding of how the identities of floral organs are established. The resulting genetic ABC program (Coen and Meyerowitz 1991), later expanded into the ABCDE model (Theißen et al., 2016 and references therein), outlines the existence of classes of gene activities, termed A, B, C, D, and E, which function in overlapping domains to determine organ identity. In this model, A + E genes specify sepals, A + B + E genes specify petals, the combination of B + C + E genes specify stamens, C + E specify carpels and C + D + E ovules. While the model was initially established based on eudicots such as *Arabidopsis*, *Antirrhinum,* and *Petunia*, it has been then evaluated in contexts beyond eudicots and monocots (Theißen and Rümpler 2018). When the focus shifted to non-model species, especially basal angiosperms, both similarities and unique characteristics became evident in relation to the model (Buzgo et al. 2004; Kim et al. 2005; Soltis et al. 2007; Yoo et al. 2010; Moschin et al. 2021).

Our results provide new data about MADS-box genes involved in flower development in early-diverging angiosperms.

We isolated from *Trithuria submersa* reproductive units 43 transcripts that have been annotated as sequences coding for MADS-box transcription factors of MIKC^C^-type. The sequences belong to ten gene sub-families: *AGAMOUS (AG)*, *PISTILLATA (PI)*, *APETALA3 (AP3)*, *B*_*sister*_* (B*_*s*_*)*, *AGL6*, *SEPALLATA (SEP)*, *AGL15*, *JOINTLESS (JNT)*, *SOC*, *AGL12*. Some of these sequences codify for MADS-box transcription factors that may belong to the A- B- C- D- E- functional classes. Among them, three AG-like (classes C/D), one AP3 and two PI (class B), one SEP (class E), and one AGL6 transcription factors have been identified. This subset of genes is quite similar to what has been found in the genome of *Nymphaea colorata* (Zhang et al. 2020) and what has been characterized in *Nymphaea caerulea* flower structures (Moschin et al. 2021), highlighting similar features between *Trithuria* and *Nymphaea* species. Moreover, in the genome of *Amborella trichopoda* two different *SEPALLATA* genes have been identified (Amborella Genome Project 2013), while in *Trithuria submersa* we found only one *SEP*, similarly to what has been obtained in *Nymphaea* species (Zhang et al. 2020; Moschin et al. 2021). No *AP1*-like sequences (class A) have been identified, even though this result is not very surprising considering that *Trithuria* flower buds possess only simple protective bracts. In addition, an *AP1*-like gene expressed in sepals and petals was also not found in *Nymphaea colorata* (Zhang et al. 2020), nor in *Nymphaea caerulea* (Moschin et al. 2021).

These findings confirm that the ABCDE model initially developed based on eudicot species, requires revisions when applied to numerous early-diverging angiosperms. More precisely, ABC homologs tend to display broader expression patterns (Kim et al. 2005; Yoo et al. 2010; Moschin et al. 2021, this work). In addition, in vitro studies demonstrated that MADS-box protein–protein interactions are more promiscuous in ANA-grade species and early-diverging eudicots, compared to core eudicots (Liu et al. 2010; Melzer et al. 2014; Rümpler et al. 2018). Therefore, it is possible to imagine a more complex scenario for early-diverging angiosperm flowers, in which more MADS-box genes have been involved. The formation of many different MADS-box protein complexes, thanks to the involvement of more genes and less strict interactions, might have been of key importance for the specialization of the varied and complex flower morphologies that we see in the ANA grade group of angiosperms.

### Characterization of the *AGAMOUS* genes in *Trithuria submersa*

*AGAMOUS* is known as a master regulatory gene that plays a key role in floral meristem determinacy and organ identity and development. In *Arabidopsis*, *AG* functions in stamen and carpel identity, floral meristem determinacy, and ovule development (Bowman et al. 1989; Mizukami and Ma 1995; Pinyopich et al. 2003).

The evolutionary history of gene duplications in the *AG* subfamily has been investigated by different studies (Becker and Theißen 2003; Jager et al. 2003; Kramer et al. 2004; Zahn et al. 2006). An ancient duplication event occurred early in angiosperm history after the divergence of the angiosperm and gymnosperm lineages, originating the *AG* and *AGL11* lineages. Kramer et al. (2004) referred to them as the C-lineage and D-lineage respectively, based on the function of specific genes in stamen and carpel identity (C-function) (Coen and Meyerowitz 1991; Schwarz-Sommer et al. 1992), and ovule development (D-function) (Angenent et al. 1995; Colombo et al. 1995, 1997, 2008). However, as orthology does not always coincide with functional equivalence, Zahn et al. (2006) chose to refer to these groups as the *AG* and *AGL11* clades. Indeed, the same authors pinpointed that it is difficult to separate D-function from C-function by phylogenetic analysis, and highlighted the amount of overlap, lability in expression and function of the *AG* subfamily among the *AG* and *AGL11* lineages (Zahn et al. 2006). Another duplication event occurred within the lower eudicots and gave rise to the *euAG* and *PLENA* (*PLE*) clades, which include *AG* and *SHATTERPROOF1* and *SHATTERPROOF2* (*SHP1/2*), respectively. Further more recent duplications occurred later within angiosperm evolution (Becker and Theißen 2003; Irish 2003; Kramer et al. 2004; Zahn et al. 2006).

Therefore, in *Arabidopsis*, a reference plant for many genetic studies, the *AG* subfamily members are *AG*, *SHP1* and *SHP2*, of the *AG* lineage, and *SEEDSTICK* (*STK*), of the *AGL11* lineage.

Given the key importance of *AG*-like genes during flower organ initiation and development and their interesting evolutionary history, a focus on *T. submersa AG* genes appears particularly interesting. Our phylogenetic analysis has demonstrated that two sequences (TsAG1 and TsAG2) are more similar to each other and fall into the AGAMOUS group of Nymphaeaceae, while the other one (TsAG3) falls into the SEEDSTICK group (or STK-like) of Nymphaeaceae.

The expression analysis of the three genes seems consistent with the phylogenetic characterization of the sequences because the two *TsAG1* and *TsAG2* share a similar expression pattern and are present in both bracts and reproductive organs, while *TsAG3* has been mainly found in female structures, especially in young ovules. These findings suggest that *TsAG1* and *TsAG2* can be considered *AG-*like genes, and *TsAG3* can probably function as an *STK*-like gene.

Genes often have a complex history: they duplicate into paralogs, sometimes acquiring new functions (neofunctionalization) or splitting the ancestral function between the paralogs (subfunctionalization) (Kirkpatrick and Barton 2006). In the case of *TsAG1* and *TsAG2,* they may share most of their functions, based on their partial overlapping expression patterns, with a specific involvement of *TsAG2* in the anthers. Conversely, *TsAG3*, grouped with other *STK-*like sequences in the phylogenetic study, and showed a specific expression domain in ovules, and it may thus belong to the *AGL11* lineage. Characterization of *AGAMOUS* genes in *Nymphaea* species has provided similar results. For example, in *Nymphaea caerulea* two different *AGAMOUS* genes share similar expression profiles, even if they seem to have acquired some functional specialization, and a third one has been identified as a *STK-*like gene (Moschin et al. 2021). Yoo et al. (2010) also identified two *AG*-like genes and an *STK*-like gene in *Nymphaea odorata*, and these findings are consistent with the published genomic data of *Nymphaea colorata* (Zhang et al. 2020). Altogether, these results support the idea that in Nymphaeales the *AGAMOUS* subfamily is composed of three genes, one of which belongs to the *AGL11/STK* clade, and the other two to the *AG* clade. The evolutionary history of the two *AG* paralogs might have varied in different species through sub-functionalization or neo-functionalization events, providing the genetic basis for acquiring or specializing some novelties in their reproductive development.

## Conclusions

This work provides novel data on the reproductive development of *Trithuria submersa*, an angiosperm species belonging to the family Hydatellaceae, which was placed quite recently in the water-lily order Nymphaeales (Saarela et al. 2007). Morphological studies well described the structure of the reproductive unit (Rudall et al. 2007), which appeared to us very interesting to study, especially for the characterization of some molecular players that regulate flower organ identities (i.e. *AGAMOUS* MADS-box genes).

Transcriptome analysis of flower buds has provided MADS-box transcript sequences. Among them, we characterized more deeply the three *TsAGs*, finding different expression domains during *Trithuria* flower development. In situ hybridization experiments demonstrated that *TsAG1* and *TsAG2* are expressed in different flower organs, including bracts, while *TsAG3* is more ovule-specific, probably functioning as a D-type gene. This study adds some novel data in the field of MADS-box genes of non-model species, and molecular data from early-diverging angiosperm species can greatly help to shed light on the key developmental genes that may have been very important in shaping first flower appearance and evolution.

## Supplementary Information

Below is the link to the electronic supplementary material.

Supplementary file1 (DOCX 12 KB)

Supplementary file2 (PDF 262 KB)

## Data Availability

All data supporting the findings of this study are available within the paper and its Supplementary Information. MADS-box gene sequences have been deposited in the NCBI GenBank database, and Accession Numbers are provided in Table 1.
