# Supplementary material for: Reproductive development in Trithuria submersa (Hydatellaceae: Nymphaeales): the involvement of AGAMOUS-like genes
Source: Planta. 2024 Sep 26;260(5):106. doi: 10.1007/s00425-024-04537-5 (PMC11427499; doi:10.1007/s00425-024-04537-5)
Supplement: Supplementary file 1 — Supplementary file1 (DOCX 12 KB) [file 425_2024_4537_MOESM1_ESM.docx]

**SUPPLEMENTARY MATERIALS**

**Supplementary Table 1. List of the primer sequences used in this work.**

| Primer ID | 5’ 🡪3’ Sequence |
| --- | --- |
| TsAG1_FOR | ACCAAATGAACGCCGAAGTG |
| TsAG1_REV | GGACCATGCGAAAGTTCACC |
| T7_ TsAG1_FOR | CTAATACGACTCACTATAGGGACCAAATGAACGCCGAAGTG |
| T7_ TsAG1_REV | CTAATACGACTCACTATAGGGGGACCATGCGAAAGTTCACC |
| TsAG2_FOR | GCACCAAGCTGAGAGGACTG |
| TsAG2_REV | GGTGACACTGCTTGGAAGTC |
| T7_ TsAG2_FOR | CTAATACGACTCACTATAGGGGCACCAAGCTGAGAGGACTG |
| T7_ TsAG2_REV | CTAATACGACTCACTATAGGGGGTGACACTGCTTGGAAGTC |
| TsAG3_FOR | ACTTGACGGGGGATGGACT |
| TsAG3_REV | ATGGCAACACACACCACAACAA |
| T7_ TsAG3_FOR | CTAATACGACTCACTATAGGGACTTGACGGGGGATGGACT |
| T7_ TsAG3_REV | CTAATACGACTCACTATAGGGATGGCAACACACACCACAACAA |
