# Supplementary material for: Reproductive development in Trithuria submersa (Hydatellaceae: Nymphaeales): the involvement of AGAMOUS-like genes
Source: Planta. 2024 Sep 26;260(5):106. doi: 10.1007/s00425-024-04537-5 (PMC11427499; doi:10.1007/s00425-024-04537-5)
Supplement: Supplementary file 2 — Supplementary file2 (PDF 262 KB) [file 425_2024_4537_MOESM2_ESM.pdf]

NATIONAL HERBARIUM OF VICTORIA (MEL)
MELBOURNE, AUSTRALIA

MEL 2388684

Trithuria submersa Hook.f.
Det.: Walsh, N.G., 13 Oct. 2015

Coll.: Walsh, N.G. 8347 **Date:** 13 Oct. 2015
Addit. Coll.: Schuster, T.M.; Murphy, D.J.; Holmes, G.D.

Australia: Victoria
Loc.: Black Range, Stubgate Rd, 2 km E of Mt Talbot Rd, 13 km WSW from Brimpaen.
37° 03' 33" S 142° 04' 07" E. Alt.: 205 m.
Habitat: Seasonally wet depression with fringing heathland. Moist sandy soil.
Associated taxa: Kunzea parvifolia, Drosera glanduligera, Centrolepis strigosa, C. aristata, Aphelia pumilio, Blennospora drummondii, Quinetia urvillei, *Isolepis hystrix.
Descriptive notes: Minute reddish tufts.

354. Hydatellaceae – M. Monocots – Main collection (5. Australasia)

Printed from MELISR, 30 Oct. 2015
